# Long-Term Effects of Family Resilience on the Subjective Well-Being of Offspring in the National Longitudinal Lesbian Family Study

**DOI:** 10.3390/ijerph20065149

**Published:** 2023-03-15

**Authors:** Henny M. W. Bos, Nicola Carone, Esther D. Rothblum, Audrey S. Koh, Nanette K. Gartrell

**Affiliations:** 1 Research Institute of Child Development and Education, Faculty of Social and Behavioral Sciences, University of Amsterdam, 1012 WX Amsterdam, The Netherlands; 2 Department of Brain and Behavioral Sciences, University of Pavia, 27100 Pavia, Italy; 3 Williams Institute, Los Angeles School of Law, University of California, Los Angeles, CA 90095, USA; 4 Department of Women’s Studies, San Diego State University, San Diego, CA 92182, USA; 5 Department of Obstetrics, Gynecology and Reproductive Sciences, School of Medicine, University of California, San Francisco, CA 94115, USA

**Keywords:** family resilience, subjective well-being, protective factors, offspring of sexual minority parents, homophobic stigmatization long-term effects

## Abstract

The current study used a family resilience approach to investigate why some offspring of sexual minority parents thrive despite homophobic stigmatization while others do not. Specifically, the study explored the role of two specific family functioning mechanisms (i.e., during adolescence, disclosure of offspring’s personal life to their parents, and family compatibility) in the association between experienced homophobic stigmatization at age 17 and subjective well-being at age 25, among 71 offspring (37 females and 34 males, all cisgender) of the National Longitudinal Lesbian Family Study (NLLFS). The results showed that, overall, the offspring reported healthy subjective well-being as emerging adults. However, among NLLFS offspring with less family compatibility as adolescents, homophobic stigmatization was related to higher scores on negative affect when they were emerging adults. Psychological counseling that supports adolescent-parent communication may help prevent the negative effect of homophobic stigmatization on the subjective well-being of offspring with sexual minority parents.

## 1. Introduction

Research over the past several decades has found that sexual minority parents (SMPs) are successful in their parenting practices, rearing offspring who are psychologically healthy [1,2]. These findings are particularly impressive given that SMP families are often confronted with heteronormativity and homophobic stigmatization [3]. The current article focuses on understanding why some offspring in SMP families develop resilience despite experiencing stigma and discrimination. This study aims to investigate which family functioning processes might influence the long-term adverse effects of homophobic stigmatization on the offspring’s subjective well-being (i.e., negative and positive affect).

### 1.1. Homophobic Stigma-Based Bullying of SMP Families

A recent meta-analysis showed that children and adolescents who grow up in SMP families are not generally at risk of developing adverse outcomes [4]. However, in some cases, parents and offspring in these families find it challenging to navigate stigma-based bullying [5]. For example, findings based on the U.S. National Longitudinal Lesbian Family Study (NLLFS), which is the longest ongoing, prospective study on SMP families, illustrated that both parents and children faced this specific form of stigma-based bullying due to the parents’ sexual orientation [6]. Similar results were found in other U.S. studies [7,8,9] and other countries such as Italy [10], the Netherlands [11], and Belgium [12]. Experiences of homophobic bullying varied with age: 18% of NLLFS offspring had experienced homophobic stigmatization by age 5, 43% by age 10, and 41% by age 17 [13,14,15]. Thus, the offspring’s developmental level might influence the extent to which they perceive and interpret stigma, such that young children may be less capable than adolescents of understanding that their peers are bullying them based on their parents’ sexual identity [16].

### 1.2. Sexual Minority Stress Theory and Family Stress Theory

According to the sexual minority stress model, sexual and gender minority people are exposed to unique and chronic stressors related to their marginalized status. These stressors include interpersonal prejudicial acts (e.g., negative reactions from people in their environment), as well as structural discrimination (e.g., laws permitting unequal treatment of SMPs in employment and housing) [17]. These stressors increase the potential for adverse physical and mental health outcomes in sexual minority individuals such as SMPs. In addition, although most offspring in SMP families do not identify as lesbian or gay [18], these stressors affect their mental health as they contend with stigma-based bullying due to the sexual orientation of their parents in a heteronormative society [17,19]. Studies have shown that offspring of SMP who experience this type of stigma-based bullying are more likely to have behavioral problems, lower levels of self-confidence, and more school absences during childhood and adolescence [6,10,20]. The NLLFS has also found evidence of long-term consequences of homophobic stigmatization as experienced during adolescence on behavioral problems and psychological adjustment during emerging adulthood [21,22]. As such, homophobic stigmatization may be a family stressor for some offspring in SMP families. According to the family stress theory, exposure to such stressors can lead to psychological problems for family members, especially children, when the stressors become more frequent or if there is no supportive family environment [23].

### 1.3. Family Resilience

For mental health professionals working with families who are confronted with homophobic stigmatization, it is important to understand why some offspring in SMP families are resilient in the face of family stressors, while others are not [24,25,26,27]. Cross-sectional studies carried out in the U.S. and the Netherlands found that attending schools with lesbian, gay, bisexual, and transgender curricula, having lesbian parents who participated in the lesbian community, and having frequent contact with other offspring of SMPs protected children against the negative influences of stigmatization [11,20,28,29]. However, less is known about which family functioning processes may play a role in developing resilience to adverse circumstances related to homophobic stigmatization. A resilience-oriented family approach seeks to identify, among other things, family functioning processes that are or could become critical in the positive development and well-being of offspring confronted with structural stressors [30]. According to the family resilience framework, salient processes within the family that are likely to promote resilience [31] include connectedness among family members, as well as open, transparent, and consistent communication between parents and offspring [32]. These processes may serve as protective factors against developing psychological problems when the offspring are confronted with structural stressors such as homophobic stigmatization.

### 1.4. Negative and Positive Affect

Negative affect includes feelings such as anger, sadness, and shame. Positive affect reflects the opposite and includes emotions such as being happy and enthusiastic [33,34]. Negative and positive affect are independent components of subjective well-being and are poorly correlated [32].

There is research indicating that members of ethnic minority groups who perceived current and past racial discrimination reported more negative affect [35,36,37], but less is known about the association between homophobic stigmatization and (negative and positive) affect among offspring in SMP families. In particular, there is a lack of knowledge about the long-term effects of homophobic stigmatization during adolescence when the family environment is salient [38]. Whether there is a significant association between stigma-based bullying during adolescence on well-being later in life, such as during emerging adulthood, is unknown.

### 1.5. Aim of the Current Study

Based on the above literature on minority stress, family stress, and family resilience theory, the current study focuses on which family-based protective factors might buffer the association between homophobic stigmatization and negative and positive affect among the offspring who grew up in SMP families. The research question in the present study is to examine in a cohort of offspring in SMP families whether disclosure of one’s personal life to one’s parents and family compatibility during adolescence, influenced (i.e., moderated) the association between homophobic stigmatization during adolescence and negative and positive effect during emerging adulthood.

## 2. Materials and Methods

### 2.1. The Participants

The present study is based on the 5th and 6th Waves of data collection of the NLLFS and specifically on the offspring data. As such, the study sample consisted of 71 participants (37 females and 34 males, all cisgender) who participated in both waves of data collection. All participants were conceived through donor insemination by lesbian-identified parents and were, at the time of the data collection, 17 (Wave 5) and 25 (Wave 6) years old. All participants were born in the U.S. Ninety percent of participants identified as White (*n* = 64) and 9.9% (*n* = 7) as people of color: African American/Black (*n* = 3), Latina/o or Hispanic (*n* = 1), or other/mixed (*n* = 3). A majority (87.3%, *n* = 62) had completed a bachelor’s or registered nurse degree. More than three-quarters (78.9%, *n* = 56) identified as heterosexual. Most participants lived independently from their parents (82.8%, *n* = 58), and 54.9% (*n* = 39) were involved in an ongoing relationship with a partner. Most participants (59.2%, *n* = 42) did not know their donor.

### 2.2. Sampling Procedures of the NLLFS

The present study is based on the NLLFS that started in 1986 (Wave 1) with 84 families consisting of lesbian-identified parents who were conceiving children through donor insemination [39]. Recruitment of prospective parents took place through announcements at lesbian events, women’s bookstores, and lesbian/gay publications. After Wave 1, the parents were interviewed again when their children were 2 (Wave 2), 5 (Wave 3), 10 (Wave 4), 17 (Wave 5), and 25 (Wave 6) years old. Their offspring also participated in Waves 4, 5, and 6. At Waves 4 and 5, the parents assented to the participation of their offspring, and at Wave 6 the offspring provided their own written informed consent. Data collection at Waves 5 and 6 took place through a protected online survey completed in May 2009 and October 2017, respectively. At Waves 5 and 6, each offspring who completed the online survey received a $60 gift card for their participation. The Institutional Review Board (IRB) at Sutter Health approved this study, including the protocols for Waves 5 and 6 (Project Title: The National Longitudinal Lesbian Family Study, #20.070-2; IRBNet# 348911-20). There were no deviations from the study protocols approved by this IRB. Preregistration was not required in the 1980s when the NLLFS began. More information about the NLLFS can be found on the study’s website (www.nllfs.org, accessed 1 March 2023).

### 2.3. Measures

At Wave 5, when the offspring were adolescents, data were collected about their experiences of homophobic stigmatization, disclosure of their personal life to their parents, and their feelings about family compatibility. The participants’ negative and positive affect was included in the survey at Wave 6 when the offspring were emerging adults. These Wave 5 and 6 measures were selected based on the literature regarding sexual minority stress and family resilience (Requests for the NLLFS Wave 5 and Wave 6 survey or protocol should be directed to its Principal Investigator).

#### 2.3.1. Homophobic Stigmatization, Disclosure of Personal life, and Family Compatibility Measured at Wave 5

When the offspring were 17 years old, experiences of homophobic stigmatization were measured by asking, “Have you been treated unfairly because of having a lesbian mom?” (0 = no, 1 = yes). They were also asked about disclosing their personal life to their parents with the question: “Do you feel you can confide in your mom(s) about your life?” (0 = no, 1 = sometimes, 2 = yes). Family compatibility was measured with the item, “I feel I am getting along with my parents or guardians” (0 = not at all, 10 = completely), which was derived from the Youth Quality of Life Instrument [40].

#### 2.3.2. Negative and Positive Affect at Wave 6

Two subscales from the Health Styles Survey [41] were used to assess negative and positive affect when the participants were 25 years old (Wave 6). These two subscales were originally from the Positive and Negative Affect Schedule (PANAS) scale developed by Watson et al. [33]. The PANAS is the most frequently used measure to assess negative and positive affect. Both subscales of the PANAS scales have shown excellent convergent and divergent validity and reliability scores in studies carried out in different countries [42,43].

In the PANAS the question “How often in the past 30 days have you felt?” was followed by six negative (e.g., nervous, worthless, etc.) and six positive (e.g., cheerful, in good spirits, etc.) feelings, with five answer categories for each feeling (1 = none of the time—5 = all of the time). The mean score on the six negative feelings was calculated with higher values representing higher negative affect. The same was also done for the six positive feelings for the Positive effect subscale with higher values representing higher positive affect. In the current study, Cronbach’s alpha for the Negative Affect subscale was 0.82 and for the Positive Affect subscale was 0.87.

#### 2.3.3. Demographic Information at Wave 6

At Wave 6, participants were asked about their sex assigned at birth (female or male) and gender identity (female, male, or other), whether they identified as African American or Black, Asian, Latina/o or Hispanic, Native American, Pacific Islander, White (non-Latina/o or Hispanic), other, or mixed. The Wave 6 survey also included a question about the participants’ educational level (1 = no high school diploma and no general equivalency diploma, 2 = general equivalency diploma, 3 = high school graduate, 4 = some college but no college degree, 5 = associate degree, 6 = bachelor’s or registered nurse degree, 7 = some graduate school but no graduate degree, 8 = master’s degree, 9 = doctoral or law degree, 10 = other education). Regarding sexual orientation, Wave 6 participants were asked: “Do you think of yourself as …” (1 = heterosexual or straight, 2 = lesbian, gay, or homosexual, or 3 = bisexual). Participants were asked whether they lived with their parents (0 = no, 1 = yes) and whether they were in an ongoing relationship (0 = no, 1 = yes). Finally, they were also asked to specify the type of donor used for their conception (1 = permanently unknown, 2 = open-identity but not yet met, 3 = known donor since childhood, 4 = open-identity and met him after turning 18).

Race/ethnicity, education, sexual orientation, and donor status were recorded because of the small sample sizes in the answer categories on these variables. Race/ethnicity was recoded into two categories: *People of color* (African American or Black, Asian, Latina/o or Hispanic, Native American, Pacific Islander, other, or mixed) and *White*. Education was recorded into *No associate degree* (no high school diploma and no general equivalency diploma, general equivalency diploma, high school graduate, some college but no college degree) and *Associate degree or higher* (associate degree, bachelor’s or registered nurse degree, some graduate school but no graduate degree, master’s degree, doctoral or law degree). None of the participants checked “other education.” Participants who identified as lesbian/gay/homosexual or bisexual were also pooled. Donor type was recoded into *Unknown donor* (anonymous, and open-identity but not yet met) and *Known donor* (known since childhood, and open-identity—met after turning 18).

### 2.4. Analyses

Descriptive statistics were used for the NLLFS offspring’s scores on experienced homophobic stigmatization, disclosure of personal life to parents, family compatibility, and negative and positive affect. Preliminary analyses were carried out to ensure that the demographics of the participants were not confounding variables in the effect of experienced homophobic stigmatization on negative and positive affect. For these preliminary analyses, *t*-tests were used to assess differences in sex, relationship status, and donor status on the outcome variables’ negative and positive affect. Nonparametric tests (Mann-Whitney *U*) were employed to assess the association of race/ethnicity, education, sexual orientation, and living with parents on negative and positive affect. Mann-Whitney *U* tests were computed for these demographics because the cell sizes for the categories of these variables were too small to use standard t-testing. Bivariate associations between studied variables were also computed for descriptive analyses: Pearson *r* correlations were used for the association between continuous variables and Spearman *rho* for the associations between a categorial and continuous variable. There were no missing data on the demographic variables, nor on the variables that measured experienced homophobic stigmatization, disclosure of personal life to parents, family compatibility, and negative and positive affect.

The statistical software program R [44] was used to assess whether the association between homophobic stigmatization during adolescence (i.e., predictor variable) and negative and positive affect during emerging adulthood (i.e., outcome variables) varied across the different levels of disclosure of one’s personal life to one’s parents and family compatibility during adolescence (i.e., moderator variables). The two possible moderators (disclosure of one’s personal life to one’s parents and family compatibility) were entered simultaneously in a regression analysis. First, this regression analysis was carried out for negative effect, and then for positive effect as the outcome variable. To reduce multicollinearity [45,46], the continuous variables in our analyses (disclosure of one’s personal life to one’s parents, family compatibility, and negative and positive affect) were centered around the mean scores. Significant interactions between homophobic stigmatization and disclosure, and homophobic stigmatization and family compatibility, in predicting negative or positive affect were considered as evidence for moderation. If an interaction was significantly associated with an outcome variable, the Johnson-Neyman technique was used for understanding the direction of such a significant interaction [47,48]. More specifically, the Johnson-Neyman technique allowed us to identify the range of values in which the potential moderator(s) (i.e., disclosure and family compatibility) influenced the effect of the predictor (i.e., homophobic stigmatization) on the outcome variables (i.e., negative and positive affect) [45,47].

Demographic variables that were significantly associated with negative or positive affect in the preliminary analyses were included as covariates in the moderation analyses.

## 3. Results

### 3.1. Preliminary Analyses

Of the 71 NLLFS offspring, 40.8% (*n* = 29) reported as adolescents (Wave 5) that they had been treated unfairly because of having (a) lesbian parent(s). The mean score for disclosure of their personal life to their parents during that phase of their lives was 1.28 (*SD* = 0.70; range 0–2) on a scale from 0 to 2. For family compatibility, also measured at Wave 5, the mean score was 8.18 (*SD* = 1.74; range 3–10) on a scale from 0 to 10. When the offspring were emerging adults (Wave 6), the mean scores for negative and positive affect were 2.03 (*SD* = 0.59; range 1–4.17) and 3.53 (*SD* = 0.57; range 2.50–5.00), respectively, on a scale from 1 to 5. As shown in Table 1, regarding demographic variables, only educational level was significantly related to the outcome variable negative affect, with offspring who did not have an associate degree reporting significantly higher scores on negative affect.

Table 2 shows the bivariate associations between the studied variables (i.e., homophobic stigmatization, disclosure of personal life to parents, family compatibility, and negative and positive affect). Spearman *rho* correlations showed a significant association between homophobic stigmatization and offspring’s disclosure of personal life to their parents (both measured at Wave 5). An additional analysis of variance (ANOVA) showed that participants who reported homophobic stigmatization in adolescence reported lower scores on disclosure of their personal life to their parent(s) (*M* = 1.00, *SD* = 0.66) compared to those who did not experience this type of stigmatization (*M* = 1.48, *SD* = 0.67), *F* (1, 69) = 8.81, *p* = 0.004. Furthermore, the results indicated that family compatibility during adolescence was significantly positively related to disclosing one’s personal life to parents during adolescence. High scores on family compatibility were related to high levels of disclosure of personal life to parents. Finally, lower scores on the negative affect subscale were significantly correlated with higher scores on the positive affect subscale (both measured at Wave 6).

### 3.2. Moderating Effect of Disclosure of Personal Life to Parents and Family Compatibility on the Association between Homophobic Stigmatization and Negative and Positive Effect

#### 3.2.1. Negative Affect

The regression analysis revealed that the interaction between homophobic stigmatization and disclosure of one’s personal life to one’s parents during adolescence was not significant for negative affect in emerging adulthood, while the interaction between homophobic stigmatization and family compatibility during adolescence was significant (see Table 3). In this regression analysis, education was included as a control variable because it was significantly associated with negative affect in the preliminary bivariate analyses. However, this significant association of education level disappeared after including homophobic stigmatization, personal life disclosure, and family compatibility in the multiple regression analysis.

**Figure 1:**
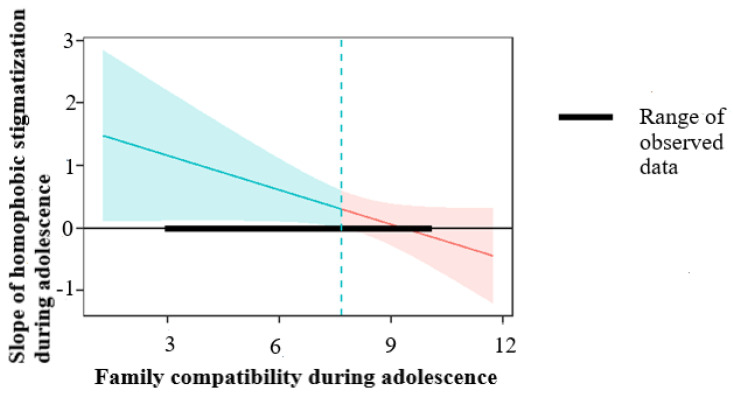
Johnson-Neyman plot. Note. The area on the left side of the dashed line is significant at *p* < 0.05.

To interpret the interaction between homophobic stigmatization and family compatibility in the findings, we used the Johnson-Neyman technique. The follow-up Johnson-Neyman analysis showed that the region of the significance of family compatibility on negative affect ranged from −9.35 (lower band) to 7.67 (upper band), indicating that any given simple slope outside this range was statistically significant. Since the family compatibility scores ranged from 3 to 10, and the interactive term was negatively associated with the outcome, one may conclude that only offspring who during adolescence experienced homophobic stigmatization and also perceived lower family compatibility reported higher negative affect during emerging adulthood. Figure 1 presents a graphic representation of this finding.

#### 3.2.2. Positive Affect

As shown in Table 4, the interaction between homophobic stigmatization and disclosure of one’s personal life to one’s parents during adolescence was not significant for positive affect as the outcome variable, nor was the interaction between homophobic stigmatization and family compatibility during adolescence. These findings indicate that the effects of homophobic stigmatization during adolescence on positive affect during emerging adulthood do not depend on either disclosure of one’s personal life to one’s parents or family compatibility during adolescence.

## 4. Discussion

The present study examined the long-term protective role of disclosure of personal life to parents and feelings of compatibility (both as components of family processes mechanisms) experienced by offspring in NLLFS families during their adolescence. This is the first time that the protective mechanism of family process components in SMP families has been studied in the association between homophobic stigmatization experienced during adolescence and subjective well-being (negative and positive affect) during emerging adulthood.

It is relevant to note that the NLLFS adult offspring reported healthy subjective well-being as evidenced by their relatively low scores on negative affect and relatively high scores on positive affect. However, when they were adolescents, about 40% reported being treated unfairly because of having (a) lesbian parent(s). More recent data on stigmatization experienced by children in SMP families (e.g., heterosexism, public outing by others, and teasing because of their family type) found that 57% reported such experiences [7]. Despite cultural changes that are more affirming of sexual and gender minorities [49], homophobic stigmatization is still an issue with which SMPs and their children contend, especially during adolescence.

The present study found that the association between experienced homophobic stigmatization during adolescence and subjective well-being was significant only in specific family process circumstances, that is, only for offspring with a lower score on family compatibility and only for the outcome variable of negative affect. Under such circumstances, whether the adolescent offspring experienced family coherence played a role in whether homophobic stigmatization had a long-term effect on their negative affect. In this vein, we found evidence of family resilience [30,31,32,50] and support for the concept that family relatedness is a basic contributor to psychological well-being [51]. Some authors assume that negative events are specifically associated with negative affect, and not with positive affect [52], which could be an explanation for the lack of significant evidence of the protective mechanisms of family compatibility in the association between experienced homophobic stigmatization during adolescence and positive affect during emerging adulthood.

The results also showed that in adolescence—a time when friendship gains importance—the family environment is still a proximal factor [53] for the offspring’s long-term development. Family compatibility is a family resilience characteristic [32,54,55] that can help SMP families counteract the adverse long-term consequences of homophobic stigmatization on the offspring’s well-being.

In the present study, we also investigated whether disclosing personal life to parents during adolescence moderated the association between experienced homophobic stigmatization during that life stage and subjective well-being as emerging adults. No significant interaction between homophobic stigmatization and disclosure was found on negative and positive affect. Thus, for the NLLFS offspring, disclosing personal life to parents was not a protective factor. It is possible that some adolescents might perceive support from their parents as intrusive [22,56], and as such, may conceal details of their personal life from their parents. It is also possible that the NLLFS offspring tried to protect their parents from finding out that they were bullied because of their parent’s sexual orientation.

Several limitations of the present study should be considered when interpreting the results. First, three variables (experienced homophobic stigmatization, disclosure of personal life to the parents, and family compatibility) in this study were measured with a one-item question, while only the outcome variables of negative and positive affect were measured with a standardized instrument. However, in large cohort studies about parenting and family diversity with a more general focus, it is not uncommon to include single-item questions [57]. Second, the NLLFS data were based on a U.S. cohort of first-generation, planned lesbian-parent families with donor-conceived offspring. The NLLFS parents were the first generation that could access reproductive technologies (ARTs) because sperm banks became available for SMP in the mid-1980s; however, the procedure was expensive and, as such, primarily utilized by those with middle to upper-income levels. To the extent that the availability of adequate economic resources promotes healthy development and adjustment in children [50,58,59], the NLLFS parental SES may have contributed to the relatively healthy subjective well-being in terms of low negative affect scores and high positive affect scores that were found in the current study. Furthermore, the NLLFS parents originally lived primarily in Boston, Washington, D.C., and San Francisco, and their experiences may not reflect those of other SMP families in the U.S. or outside the U. S. [60]. Studies within the U.S., for example, show that sexual minorities living in states with fewer legal protections have higher levels of mental health problems than those living elsewhere [61,62]. Third, the data used in this study were gathered using online questionnaires completed by only one source—the offspring. Although the NLLFS parents also completed questionnaires during Waves 5 and 6, their surveys did not include concepts of family mechanisms. Therefore, it was impossible to determine how the offspring’s perceptions of family functioning during adolescence (i.e., offspring’s disclosure of personal life to the parents and family compatibility) compared with their parents’ views. Finally, resilience, or the capacity for adjustment and achieving good outcomes in the face of adverse events such as structural experiences of homophobic stigmatization, can occur at various levels, including the individual, family, and community [26,27,32]. Future research on family resilience among SMP families should be based on larger sample sizes and have a more heterogeneous demographic sample.

Despite these limitations, the findings provide some direction for counseling sexual minority parent families that experience homophobic stigmatization. In the present study, the long-term effect of such incidents was only significantly related to negative affect when feelings of family compatibility were low. In helping these offspring, psychological counseling should not only focus on the offspring’s individual experiences but should also include parents to help them create an environment where all family members feel emotionally connected to each other. For example, parents may benefit from guidance on teaching children how to reframe experiences of homophobic stigmatization, and modeling responses that enable the children to feel more empowered.

## 5. Conclusions

By conducting a longitudinal investigation based on a family resilience approach, we found that homophobic stigmatization can have long-term adverse effects on the offspring’s well-being. Psychological counseling should focus on the circumstances that contribute to this vulnerability and should be embedded in the family context.

## Figures and Tables

**Table 1:** Comparisons of sex, race/ethnicity, education, sexual orientation, living with parents, relationship status, and donor type with negative and positive affect (measured at Wave 6) (N = 71).

	Negative Affect			Positive Affect		
	*M* (*SD*)	*t*/Mann-Whitney *U* Test	*p*	*M* (*SD*)	*t*/Mann-Whitney *U* Test	*p*
Sex assigned at birth		−0.05	0.961		1.44	0.153
Female	2.02 (0.63)			3.63 (0.55)		
Male	2.03 (0.56)			3.43 (0.58)		
Race/ethnicity		168.0	0.276		183.5	0.432
People of color	1.79 (0.34)			3.64 (0.38)		
White people	2.05 (0.61)			3.52 (0.59)		
Education		162.5	0.042		215.0	0.266
No associate degree	2.57 (0.99)			3.39 (0.79)		
Associate degree or higher	1.95 (0.48)			3.55 (0.54)		
Sexual orientation		368.5	0.464		406.5	0.848
Heterosexual	1.99 (0.57)			3.53 (0.54)		
Bisexual/Gay/Lesbian	2.16 (0.68)			3.53 (0.69)		
Living with parents		254.0	0.141		235.0	0.077
No	1.98 (0.53)			3.58 (0.50)		
Yes	2.28 (0.82)			3.35 (0.84)		
Relationship status		1.66	0.104		−1.18	0.242
No ongoing relation	2.16 (0.77)			3.44 (0.64)		
Ongoing relation	1.91 (0.38)			3.61 (0.50)		
Donor type		0.30	0.763		0.429	0.669
Unknown donor	2.04 (0.60)			3.56 (0.65)		
Known donor	2.00 (0.59)			3.50 (0.44)		

Note: *t*-tests were carried out for gender, relationship status, and donor type; Mann-Whitney *U* tests for race/ethnicity, education, sexual orientation, and living with parents. *p* < 0.05 indicates significant findings.

**Table 2:** Percentage (*n*), means (*M*), standard deviations (*SD*), observed and expected values, and intercorrelations on the studied variables (N = 71).

	% (*n*)/ *M* (*SD*)	Observed Values [Expected Values]	1.	2.	3.	4.
1. Homophobic stigmatization at Wave 5, yes	40.8 (29)	-	-			
2. Disclosure of personal life to parents at Wave 5	1.28 (0.70)	0–2 [0–2]	−0.35 *	-		
3. Family compatibility at Wave 5	8.20 (1.74)	3–10 [0–10]	−0.17	0.53 **	-	
4. Negative affect	2.03 (0.59)	1–4.17 [0–5]	0.21	−0.06	−0.12	-
5. Positive affect	3.53 (0.57)	2.50–5.00 [0–5]	−0.19	0.09	0.13	−0.46 **

Note: All correlations with homophobic stigmatizations are calculated with a Spearman *rho*, whereas a Spearman *r* was used for the other correlations. * *p* < 0.001, ** *p* < 0.001.

**Table 3:** The moderating effect of disclosing personal life to parents and family compatibility during adolescence (Wave 5) on the relationship between homophobic stigmatization during adolescence (Wave 5) and negative affect during emerging adulthood (Wave 6).

					95% Confidence Intervals (CIs)	
	Estimate	SE	*t*	*p*	Low	High
Control variable:						
Level of education at Wave 6	−0.54	0.31	−1.71	0.093	−1.17	0.09
Predictor variable:						
Homophobic stigmatization during adolescence (Wave 5) (A)	0.21	0.15	1.36	0.178	−0.10	0.51
Family functioning mechanisms variables:						
Disclosing personal life to parents during adolescence (Wave 5) (B)	−0.20	0.12	−1.70	0.093	−0.44	0.03
Family compatibility during adolescence (Wave 5) (C)	0.09	0.05	1.88	0.065	−0.01	0.18
Interaction between predictor and family functioning mechanisms variables:						
A*B	0.36	0.21	1.74	0.086	−0.05	0.78
A*C	−0.18	0.08	−2.19	0.032	−0.35	−0.02
	R^2^ = 0.21, *F* (6, 64) = 2.77, *p* = 0.047					

Note: Disclosing personal life to parents (B) has a moderating effect on the association between homophobic stigmatization (A) and negative affect (the outcome variable) when the interaction between disclosure and homophobic stigmatization (A*B) is significant at *p* < 0.05. Family compatibility (C) has a moderating effect on the association between homophobic stigmatization (A) and negative affect (the outcome variable) when the interaction between family compatibility and homophobic stigmatization (A*C) is significant at *p* < 0.05.

**Table 4:** The moderating effect of disclosing personal life to parents and family compatibility during adolescence (Wave 5) on the relationship between homophobic stigmatization during adolescence (Wave 5) and positive affect during emerging adulthood (Wave 6).

					95% Confidence Intervals (CIs)	
	Estimate	SE	*t*	*p*	Low	High
Predictor variable:						
Homophobic stigmatization during adolescence (Wave 5) (A)	−0.20	0.13	−-1.52	0.133	−0.48	0.06
Family functioning mechanisms variables:						
Disclosing personal life to parents during adolescence (Wave 5) (B)	0.18	0.16	1.19	0.238	−0.13	0.51
Family compatibility during adolescence (Wave 5) (C)	−0.07	0.07	−1.09	0.281	−0.20	0.06
Interaction between predictor and family functioning mechanisms variables:						
A*B	−0.32	0.21	−1.49	0.140	−0.74	0.11
A*C	0.16	0.08	1.91	0.061	−0.01	0.32
	R^2^ = 0.08, *F* (5, 65) = 1.98, *p* = 0.102					

Note: Disclosing personal life to parents (B) has a moderating effect on the association between homophobic stigmatization (A) and positive affect (the outcome variable) when the interaction between disclosure and homophobic stigmatization (A*B) is significant at *p* < 0.05. Family compatibility (C) has a moderating effect on the association between homophobic stigmatization (A) and positive affect (the outcome variable) when the interaction between family compatibility and homophobic stigmatization (A*C) is significant at *p* < 0.05.

## Data Availability

The data reported in this article are not publicly available and this study was not preregistered. Requests to access the dataset should be directed to the Principal Investigator of the NLLFS.
